# Nuclear Data Evaluation for Mass Chain A=217:Odd-Proton Nuclei

**DOI:** 10.1371/journal.pone.0146182

**Published:** 2016-01-13

**Authors:** Sherif S. Nafee, Salem A. Shaheen, Amir M. Al-Ramady

**Affiliations:** 1 Physics Department, Faculty of Science, King Abdulaziz University, Jeddah, 20589, Saudi Arabia; 2 Physics Department, Faculty of Science, Alexandria University, Alexandria, 21221, Egypt; 3 Deanship of Graduate Studies, King Abdulaziz University, Jeddah, 20589, Saudi Arabia; University of Maribor, SLOVENIA

## Abstract

Thallium (Tl81217), Bismuth (Bi83217), Astatine (At85217), Francium (Fr87217), Actinium (Ac89217) and Protactinium (Pa91217) are of odd-proton numbers among the mass chain A = 217. In the present work, the half-lives and gamma transitions for the six nuclei have been studied and adopted based on the recently published interactions or unevaluated nuclear data sets XUNDL. The Q (α) has been updated based on the recent published work of the Atomic Mass Evaluation AME2012 as well. Moreover, the total conversion electrons as well as the K-Shell to L-Shell, L-Shell to M-Shell and L-Shell to N-Shell Conversion Electron Ratios have been calculated using BrIcc code v2.3. An updated skeleton decay scheme for each of the above nuclei has been presented here. The decay hindrance factors (HF) calculated using the ALPHAD program, which is available from Brookhaven National Laboratory’s website, have been calculated for the α- decay data sets for ^221^Fr-, ^221^Ac- and ^221^Pa- α-decays.

## Introduction

Alvarez-Pol et al., [1] identified ^217^Tl from the ^9^Be(^238^U, x) reaction when a 1 GeV/nucleon beam from the SIS18 synchrotron at the Gesellschaft für Schwerionenforschung (GSI), Germany at an intensity of 1.5 ×10^9^ ions/spill bombarded a ^9^Be target of 2500 gm/cm^2^. The ^217^Tl isotope was separated by means of a high resolving power magnetic spectrometer Fragment Separator (FRS). Two plastic scintillators and two multisampling ionization chambers were used to identify the nuclide based on the magnetic rigidity, time–of -flight, energy loss and atomic number. However, the discovery of the ^217^Bi isotope was attributed to Pfützner et al., [2] using the same facility. The spectrum was investigated by means of γ-γ, α-γ coincidence and spectrum-multiscaling measurements [3]. The RISING array of 15 Ge clusters was used to detect the γ- rays. Each cluster has seven elements.

Fry and Thoennessen [4] reported that thirty–nine isotopes of Astatine (At) have been discovered based on the Hartree-Fock-Bogoliubov model (HFB-14). Meanwhile, the discovery of ^217^At was reported in 1947 by Hagemann et al., [5] and English et al., [6], by studying the decay series (4n+1) of ^233^U. The half-life was reported to be 18 ms.

Hahn et al., [7] reported the observation of ^217^Fr through the decay of ^229^Np produced in ^233^U(p, 5n) reactions in which a beam of protons of 32–41.6 MeV bombarded an enriched ^233^U target in the Oak Ridge Isochronous Cyclotron. The α emissions were measured by a surface–barrier Si(Au) detector. The measured α was reported to be 8.31±0.02 MeV.

Valli and Hyde [8] observed the ^217^Pa in 1968 through (6n) and (1p8n) fusion-evaporation reactions. In these reactions ^203^Tl and ^206^Pb targets were bombarded by 166 MeV ^20^Ne beams from the Berkeley HILAC. The recoils were deposited on a metallic surface in front of a semiconductor detector with a helium gas jet recoil transport apparatus [9]. The adopted half-life by Akovali [10] was 3.48(9) ms. Several years later, in 1972, Nomura et al., reported the observation of ^217^Ac through a (5n) fusion–evaporation reaction in which a 91 MeV ^14^N beam from the RIKEN IPCR cyclotron bombarded a ^208^Pb target [11]. Alpha-particle spectra were measured with a surface-barrier Si detector. The measured half-life for the ^217^Ac was 0.10± 0.01μs, whereas, the adopted one by Akovali [10] was 69(4) ns.

The latest nuclear decay data evaluations for the above nuclides were carried out by Akovali in 2003 [10]. The reported half- lives for ^217^Bi,^217^At,^217^Fr,^217^Ac and ^217^Pa, were 93(3) s, 32.3(4) ms, 19(3) μs, 69(4) ns and 3.6(8) ms, respectively. There was no record for ^217^Tl in 2003. An updated evaluation for ^217^Tl was in 2011, whereas, for ^217^Bi it was in 2014, both of which are available at Brookhaven National Laboratory's website: www.nndc.bnl.gov. This paper presents the results of the evaluations of the odd-proton nuclei among the members of the mass chain A = 217 (^217^Tl, ^217^Bi, ^217^At), ^217^Fr, ^217^Ac and ^217^Pa), which have been performed in the frame of the KASCT Research Contract no. 11-MAT2037-03, using the procedures adopted by the DDEP working group. The references cut-off date was 2015, March 31. The calculated and adopted parameters will be used to update the Evaluated Nuclear Structure and Decay Data Files (ENSDF) for those nuclides under consideration, which were appraised in 2003. The complete and updated datasets for all nuclides are of great importance for the development of different aspects of nuclear technologies.

### Procedure for Decay Data Evaluation

The half-life of ^217^At was measured using the ion-implanted technique by measuring α- and β- particles from weak ^225^Ac sources [12]. The decay series of the ^225^Ac was studied by a 900 mm^2^ Canberra Passivated Implanted Planar Silicon (PIPS) detector in a quasi 2π counting system. Recoils from ^225^Ac were collected to measure the half-life of ^221^Fr, which is the parent of ^217^At. It was reported that the possible configuration for ^217^At in analogy to ^215^At is ((π h_9/2_)^+2^(π f_7/2_)(ν g_9/2_)^-4^) [13, 14].

Actinium-217 was produced from the ^221^Pa α- decay [15] and from the (HI, xnγ) reactions such as ^205^Tl (^16^O, 4n), ^206^Pb (^15^N, 4n) and ^209^Bi (^12^C, 4n) reactions using a 96 MeV ^16^O, 80 MeV ^15^N and 75 MeV ^12^C beams [16]. The half-life of ^217^Ac was deduced from alpha-gamma (αγ), gamma-gamma (γγ), alpha-conversion electron (α-ce) and (ce-ce) coincidence experiments. Whereas, ^217^Fr was produced from ^221^Ac α- decay or from ^210^Pb (^11^B, 4nγ) using a ^11^B beam of energy ranges from 52 to 68 MeV [17]. The measured spectrum has been studied using the γ-γ coincidence techniques.

The calculation of the hindrance factor(s) of β^—^decay or the so-called log *ft* value was carried out for the direct feeding(s) to the excited states in the β^—^decay. The electron capture (ε) decays have generally been computed by the evaluator from the I(γ+ce) intensity balances at each level. The log *ft* values describe the shape of the spectrum and can be discussed as follows.

The total decay constant λ for a constant nuclear matrix element η is given as:

$$\lambda =\frac{g^{2}\eta^{2}m_{e}^{5}C^{4}}{2\pi^{3}{ћ}^{7}}\;f\left(Z,\;Q\right)$$ (1)

where, ƒ(Z,Q) is a Fermi integral, which is constant for a given β^—^decay and can be calculated by numerical expressions. *g* is the strength of the weak interaction between the nucleons, electron and the neutrino which is constant and assigned as 0.88×10^−4^ MeV.fm^3^. m_e_ is the mass of the electron and C is the speed of light. And η is a constant nuclear matrix element representing the overlap between the final and initial nuclear states.

Eq 1 can be rewritten in terms of the half-life of the parent t_1/2_ as:

$$ft_{1/2}=\text{ln}\left(2\right)\frac{2\pi^{3}{ћ}^{7}}{g^{2}\eta^{2}m_{e}^{5}C^{4}}$$ (2)

The logarithm of the left hand side in Eq 2 is called log *ft*. A rapid method to calculate the log *ft* values has been reported in [18]. The β^—^decay transitions between the initial and final states can be classified based on the log *ft* values from [19, 20] in Table 1.

**Table 1:** The β^—^decay transitions between the initial and final states.

Transition Type	Log *ft*	Spin change l_β_	Parity change (Δπ)
Super-allowed	2.9–3.7	0	No
Allowed	4.4–6	0	No
First forbidden	6–10	1	Yes
Second forbidden	10–13	2	No
Third forbidden	>15	3	Yes

The hindrance factors *HF* in the α- decay are calculated by Eq 3:

$$HF_{i}=\frac{T_{1/2}^{Exp}\left(\alpha_{i}\right)}{T_{1/2}^{Theory}\left(\alpha_{i}\right)}=\frac{T_{1/2}^{Exp}/P_{i}}{T_{1/2}^{Theory}}$$ (3)

Where, $T_{1/2}^{Exp}\left(\alpha_{i}\right)\;$ is the partial half-life for the excited state having a given α—decay branching ratio *P*_*i*_ ${(T}_{1/2}^{Exp}\left(\alpha_{i}\right)\;=\;T_{\frac{1}{2}}^{Exp}/P_{i})$. All the theoretical half-life values in the present evaluation $T_{1/2}^{Theory}\left(\alpha_{i}\right)\;$ were obtained from the spin-independent equations of Preston [21]. Five classes of α- transitions were found based on the *HF* values. For the hindrance factor between 1 and 4, the transition is called a favored transition in which the emitted α- particle is assembled from two low lying pairs of nucleons in the parent nucleus, leaving an odd nucleon in its initial orbital. For hindrance factors between 4 and 10, it indicates a mixing or favorable overlap between the initial and final nuclear states. For values between 10 and 100, it indicates that the spin projections of both initial and final states are parallel, but the wave-function overlap is not favorable. For values ranging from 100 up to1000, it indicates that the transitions occur with a change in parity but with projections of initial and final states being parallel. Finally, for hindrance factors of >1000, it indicates that the transition involves a parity change and a spin flip.

The electric quadrupole transition probability B(E2: $2_{1}^{+}-0_{1}^{+}$) and the energy ratio R(4/2) = E(4^+ 1^)/E(2^+ 1^) were calculated from the proton-neutron interaction, which is proportional to the product of the number of active protons and neutrons (*NpNn*).

The associated log *ft* values, the hindrance factors, and the statistical analysis of γ–ray data and the deduced level schemes were calculated using the computer codes LOGft, ALPHAD, BrIcc, which are available at Brookhaven National Laboratory's website: www.nndc.bnl.gov. The weighted average values for half-lives were calculated when we want to calculate an average that is based on different percentage values for several categories or when we have a group of values with frequencies associated with it using the AveTool code. All associated uncertainties are expressed at the k = 1 confidence level (i.e. 68% coverage). Using level energies from measured values of energies of transitions, the GTOL code was used to determine the intensity balance. The absolute intensities of γ-rays and the normalization factor for the transferring of the relative intensities to the intensities per 100 decays of the parent nucleus have been calculated using the GABS code. In addition, the theoretical conversion coefficients were deduced from the BrIcc code: v2.3S (29–March–2011) [22] with "Frozen Orbitals" approximation, and with an implicit uncertainty of 1.4% (k = 2 confidence level). The probabilities of internal conversion are represented as conversion coefficients by Eq 4:

$$\alpha =\frac{\lambda_{e}}{\lambda_{\gamma }}$$ (4)

Where, *λ*_*e*_ and *λ*_*γ*_ are the probabilities for emission of conversion electrons and γ’s, respectively [23]. The total conversion coefficient represents the sum of the probabilities of conversion electrons in different atomic shells as in Eq 5:

$$\alpha_{T}=\alpha_{K}+\alpha_{L}+\alpha_{M}+\ldots \ldots \ldots \ldots \ldots$$ (5)

where,

$$\alpha_{K}=\frac{\lambda_{K}}{\lambda_{\gamma }},\;\alpha_{L}=\frac{\lambda_{L}}{\lambda_{\gamma }}\text{ and }\alpha_{M}=\frac{\lambda_{M}}{\lambda_{\gamma }}$$ (6)

The conversion coefficients for mixed transitions are given as a function of a mixed ratio δ as in Eq 7:

$$\alpha_{K}=\frac{\alpha_{Ml}+\delta^{2}\alpha_{E\left(l+1\right)}}{1+\delta^{2}}$$ (7)

The values of Q(β), Q(α), and the separation energies of the neutrons and the protons S_n_ and S_p_ were calculated using the 2012 Atomic Mass Evaluation code (AME2012), available from the Atomic Mass Data Center (AMDC), Institute of Modern Physics (IMP), Chinese Academy of Sciences [24].

## Results and Discussions

The Q-values, the separation energies of the neutron, the proton, the two neutrons and the two protons (S_n_, S_p_, S(2N) and S(2P), respectively, as well as their associated uncertainties were calculated using the Atomic Mass Evaluation AME2012 for Tl81217, Bi83217, At85217, Fr87217, Ac89217 and Pa91217 and listed in Table 2, respectively. All energies are expressed in keV unless otherwise noted. All associated uncertainties are expressed at the k = 1 confidence level (i.e. 68% coverage).

**Table 2:** Evaluated Q-values and separation energies of the neutrons and the protons S_n,_S_p_, S(2N) and S(2P).

	Tl81217	Bi83217	At85217	Fr87217	Ac89217	Pa91217
**Q**_**β**_^**-**^	6073 SY* (499)	2845(19)	737(6)	-1573(11)	-3514(24)	-5901 SY(113)
**S**_**n**_	4476 SY (499)	5215(21)	5933(6)	6728(8)	7512(16)	8800(7)
**S**_**p**_	8835 SY (566)	6039 SY(200)	4677(5)	3227(9)	1877(14)	520(5)
**Q**_**α**_		4520(30)	7201(12)	8469(4)	9832(10)	8489(4)
Q(β-N)**	2762 SY (466)					
**S(2N)**	7741 SY (499)	9061(23)	10492(8)	12146(9)	13470(17)	16940(9)
**S(2P)**		15759(299)	11831(16)	9008(9)	6193(13)	3540(5)
Q(ECP)***						1640(5)

* SY means deduced from systematic trend.

** is β-decay followed by a neutron emission Q-value.

***ECP is the electron capture followed by a proton emission.

The measured half -lives T_1/2_ and the Predicted spin-parity values J^π^ (“from systematics and calculations”) for the ground states g.s. of the nuclides under consideration are listed in Table 3.

**Table 3:** The measured half-lives T_1/2_ and predicted spin-parity J^π^ values.

	Tl81217	Bi83217	At85217	Fr87217	Ac89217	Pa91217
**T**_**1/2**_	>300 ns	98.5(13) s	32.8(3) ms	19(3) μs	69(4) ns	3.6(8) ms
**J**^**π**^	1/2+	(9/2-)	9/2-	9/2-	9/2-	9/2-
**references**	[1]	[25–27]	[12]	[10, 28–31]	[10]	[32–37]

The decay Data for the ground state g.s for ^217^Bi was only available in the previous evaluation [10]. However, new energy levels and γ- rays have been measured from the ^9^Be (^238^U,X) in [24–26]. Meanwhile, the half-life of ^217^Bi was adopted from the weighted average of the half-lives of the γ- transitions through the α decay of ^217^Po [27], which were 93(3) s for 254.1 γ, 100.5(13) s and 98(1) s for 264.4 γ [26], respectively. In Table 3, the half-life of ^217^Fr was adopted in [28] from the unweighted average measured half-lives of 22(5) μs [29], 16(2) μs [28, 30] and 15(3) μs [31], respectively. Similarly, the half-life of ^217^Pa is adopted in the present evaluation from the unweighted average of the measured half-lives of 4.9(6) ms, 3.4(2) ms, 2.3(*+5–*3) ms, 3.4(1) ms and 3.8(2) ms [32–36], respectively. Meanwhile, J^π^ was predicted from systematics and calculations in [37]. The half- life of ^217^At and its uncertainty were reported in [12].

The energy levels with their uncertainties, their spins-parities J^π^, the gamma- transition energies E_γ_, their intensities I_γ_ (%), their associated uncertainties, their assigned multipolarities (MULTI.), the internal conversion coefficients (Ice(K)), and the total internal conversion coefficients (Icc) with their associated uncertainties calculated using BrIcc v2.3S for ^217^Bi, ^217^At, ^217^Fr, ^217^Ac and ^217^Pa are listed in Tables 4–8, respectively.

**Table 4:** ^217^Bi nuclear energy levels and associated properties [3].

Energy Levels (keV)	J^π^	E_γ_ (keV)	I_γ_	MULTI.	α(K) ×10^−3^	α(L) ×10^−3^	α(M) ×10^−3^	α(N)×10^−3^	Icc ×10^−3^
0.0	(9/2-)			[E2]					
744(1)	(13/2-)	744	100	[E2]	9.66(14)	2.26(4)	0.55(8)	0.14(20)	12.7
1236(1)	(17/2-)	492	100	[M1,E2]	22.2(4)	7.33(11)	1.83(3)	0.46(7)	31.9
1429(2)	(15/2-, 17/2-)	685	100	[E2]	27.0(16)	4.9(22)	1.2(5)	0.3(13)	33.0
1436(2)	(21/2-)	200	100	[E2]	167.7(24)	209.(3)	55.0(8)	14.0(20)	449.0
1436+x*	(25/2-)	20–90							

* Uncertainties were not given by the authors.

Square brackets [] in the MULTI column are used to denote a value deduced solely from level scheme considerations, whereas, parentheses () around J^π^ values are used to indicate that the values are based on weak arguments.

**Table 5:** ^217^At nuclear energy levels and associated properties [38].

Energy Levels (keV)	J^π^	E_γ_ (keV)	I_γ_	MULTI.*	α(K)×10^−3^	α(L)×10^−3^	α(M)×10^−3^	α(N)×10^−3^	Icc×10^−3^
0.0	9/2-								
100.25(2)	7/2-	100.25(2)		M1	9.66(14)	1.76(25)	0.416(6)	0.198(16)	11.97(17)
218.12(2)	5/2-	117.82(3)	0.2(12)	M1	6.13(9)	1.10(16)	0.261(4)	0.068(10)	7.58(11)
		218.12(2)	100(2)	E2	0.138(20)	0.17(24)	0.045(7)	0.017(17)	0.367(6)
272.07(4)	3/2-	53.81(3)	16(4)	M1		10.79(16)	2.56(4)	0.662(10)	14.17(20)
		171.82(3)	100(40)	E2	0.226(4)	0.471(7)	0.126(18)	0.033(5)	0.886(12)
368.23(4)	(3/2)-	96.3(3)	15(7)	M1+ E2		3.5(16)	0.9(5)	0.23(12)	4.7(21)
		150.21(3)	100(5)	M1	3.08(5)	0.550(8)	0.130(19)	0.034(5)	3.8(6)
382.34(4)	(7/2)-	282.12(9)	21(3)	(M1+ E2)	0.30(23)	0.077(17)	0.019(4)	0.005(9)	0.41(25)
		382.34(4)	100(6)	M1	0.231(4)	0.041(6)	0.010(14)	0.0024(4)	0.284(4)
410.64(5)	(13/2)-	410.64(5)		E2	0.034(5)	0.015(22)	0.004(6)	0.001(15)	0.055(8)
424.35(7)	(5/2,7/2,9/2)-	324.10(6)		M1	0.362(5)	0.064(9)	0.015(22)	0.004(6)	0.446(7)
537.5(5)	(9/2+)	437.0(5)	19(3)						
		537.8(8)	100(10)						
568.5(3)	(7/2,9/2)	468.3(7)	100(22)						
		568.5(3)	86(29)						
577.0(5)	(7/2)-	208.3(6)	12.5(25)	M1	0.077(11)	0.013(19)	0.003(5)	0.001(12)	0.095(14)
		359.86(4)	100(5)						
		576.9(4)	7.3(10)						
652.0(2)	9/2-	652.0(2)							
664.4(2)	7/2-	282.12							
		446.30(8)							
		562.3(12)	100(9)						
		665.0(2)							
809.3(2)	5/2-	809.3(2)							
891.9(3)	3/2-	891.9(3)							

* The mixing ratio for the mixed multipolarities and the associated uncertainties δ for Eγ = 96.3 and 282.12 keV were calculated to be 0.7(7) and 1(5), respectively, from the αγ coincidence data in [38].

**Table 6:** ^217^Fr nuclear energy levels and associated properties [17].

Energy Levels (keV)	J^π^	E_γ_ (keV)*	I_γ_**	MULTI.***	α(K)×10^−3^	α(L)×10^−3^	α(M)×10^−3^	α(N)×10^−3^	Icc×10^−3^
0.0	9/2-								
209(20)									
275(15)									
363.6(3)	13/2-	363.6(3)		E2	46.6(7)	27.2(4)	7.10(11)	1.86(3)	83.2(12)
484(15)									
704.2(5)	17/2-	340.6(3)		E2	53.4(8)	34.5(5)	9.03(13)	2.37(4)	99.0(15)
1077.0(6)	21/2-	372.8(3)		E2	44.3(7)	24.9(4)	6.48(10)	1.70(25)	77.7(11)
1256.1(6)		179.1(3)							
1355.0(6)	23/2+	278.0(3)		E1	33.8(10)	5.97(18)	1.42(5)	0.369(11)	40.7(12)
1509.7(6)	25/2-	154.4(3)		E1	130.5(20)	26.0(4)	6.22(10)	16.1(24)	164.7(25)
		432.8(3)		E2	32.6(5)	14.8(21)	3.84(6)	1.0(15)	52.5(8)
1688.9(7)	(+)	334.0(3)		(E2)	55.7(5)	37.0(14)	9.70(4)	2.55(10)	106(4)
		423.6(3)		(E2)	32.6(5)	32.6(5)	3.84(6)	10.0(15)	53.6(8)
1713.8(7)	27/2+	204.0(3)							
		358.0(4)		E2	47.9(7)	28.5(4)	7.45(11)	1.95(3)	86.3(13)
1988.5(7)	29/2-	274.7(3)							
		478.8(3)		E2	26.6(4)	10.7(15)	2.74(4)	0.717(11)	40.9(6)
2111.1(8)	31/2+	122.5(3)							
		397.4(3)		E2	38.8(6)	19.9(3)	5.16(8)	1.35(20)	65.5(10)
2154.5(8)		465.6(3)							
2516.5(9)	35/2+	405.4(3)							
2582.0(9)		427.5(3)		E2	33.4(5)	15.5(22)	4.0(6)	1.05(15)	54.2(8)
2618.0(9)		507.0(3)							
3002.3(9)	39/2+	485.8(3)		E2	25.8(4)	10.2(15)	2.61(4)	0.684(10)	93.5(6)

* ^210^Pb(^11^B,4nγ) ^217^Fr.

** I_γ_s were not given by the authors in [17].

*** The Multipolarities were deduced by [17] from gamma-ray angular distributions and angular correlations. Since no delay component was observed in γγ(t), M2 multipolarities were ruled out for quadrupole transitions in ^217^Fr.

**Table 7:** ^217^Ac nuclear energy levels and associated properties [15].

Energy Levels(keV)*	J^π^	E_γ_ (keV)	I_γ_	MULTI.**	α(K)×10^−3^	α(L)×10^−3^	α(M)×10^−3^	α(N)×10^−3^	Icc×10^−3^
0.0	9/2-								
660.3(5)	13/2-	660.3		E2	15.4(22)	4.74(7)	1.19(17)	0.318(5)	21.8(3)
670.1(5)	11/2-	670.1		M1+E2	40.0(3)	9.0(5)	2.1(10)	0.6(3)	50.0(4)
1146.7(8)	17/2-	486.4		E2	27.5(4)	11.8(17)	3.05(5)	0.811(12)	43.4(6)
1149.2(10)	15/2-	478.9	100(25)	E2	27.5(4)	11.8(17)	3.05(5)	0.811(12)	43.4(6)
		489	75(25)	M1(+E2)	100(7)	21.0(10)	5.10(22)	1.40(6)	120(9)
1498.2(9)	19/2-	349	76(10)	E2	52.9(8)	36.9(6)	9.75(14)	2.59(4)	0.103(15)
		351.5	100(10)	M1+E2	310(5)	6.60(6)	16.1(12)	5.8(4)	400(6)
1528.5(9)	21/2-	381.8		E2	44.2(7)	26.7(4)	7.02(10)	1.87(3)	80.3(12)
1682.3	(23/2)-	153.8		M1+E2	3710(20)	835(14)	205(5)	74.2(19)	4830(18)
1792	(25/2)-	110		M1	10310(15)	1970(3)	0.473(7)	0.125(18)	12920(18)
1916	(27/2)-	234		E2	0.118(17)	0.175(25)	47.2(7)	12.6(18)	0.357(5)
2013	(29/2)+	96	50(10)	E1+M2	1200(9)	0.320(23)		0.120(9)	1600(12)
		220	100(20)	M2	5310(8)	1721(24)	0.444(7)	0.119(17)	7620(11)

*Uncertainties in the energy levels and in the E_γ_s were not given by the authors in [15] from the (HI,xnγ).

** The mixing ratio for the mixed multipolarities and the associated uncertainties for E_γ_ = 670.1, 489, 351.5 and 153.8 keV were <2, 0.65(20±1), 0.39(8) and 0.15(5), respectively.

**Table 8:** ^217^Pa nuclear energy levels and associated properties [10, 39–40].

Energy Levels (keV)*	J^π^	E_γ_ **(keV)	I_γ_	MULTI.***
0.0	9/2-****			
1854(7)*****	29/2+******			

* The energy level was calculated in the previous evaluation [10] from the difference between the energies of the 10157- and 8337-keV α's, which were emitted from the 1850 keV and the 0.0 states, respectively.

**No further information about γ-transitions.

*** No multipolarities were assigned and therefore BrIcc cannot be run.

****The J^π^ was measured in [39].

***** This energy level was observed by α-γ spectroscopy in [39].

******The J^π^ was measured in [40].

The α- energies, α- intensities, their associated uncertainties and the hindrance factors *HF* calculated by LOG *ft* are listed in Table 9 for ^217^At, ^217^Fr and ^217^Ac from the ^221^Fr-, ^221^Ac- and ^221^Pa- α decays, respectively.

**Table 9:** The α- energies (E_α_), α- intensities (I_α_, in %), their associated uncertainties and the hindrance factors *HF* calculated by LOG *ft* for ^217^At, ^217^Fr and ^217^Ac.

^217^At [41]				^217^Fr [42]				^217^Ac [29]			
E_α_	E_level_	I_α_	*HF*	E_α_	E_level_	I_α_	*HF*	E_α_	E_level_	I_α_	*HF*
5500(40)	891.9	0.0009(20)		7170(10)	484	≈ 2	4.4(20)	9075(30)	0.0	≤100	1.4
5530(25)	809.3	0.0009(20)		7377(10)	273	9(2)	6.2(17)				
5689(3)	664.4	0.002(1)	150(80)	7440(15)	209	21(5)	4.3(13)				
5697(4)	652	≈0.001	340	7645(10)	0.0	68(5)	6.3(11)				
5776(3)	577.5	0.06(1)	13(3)								
5783(4)	568.5	0.005(2)	170(70)								
5813(3)	537.5	0.004(2)	300(16)								
5925(3)	424.3	0.03(1)	140(50)								
5938.9(20)	410.6	0.17(3)	28(5)								
5965.9(25)	382.3	0.08(1)	79(11)								
5979.9(20)	368.2	0.49(3)	15(2)								
6037(3)	310.3	0.003(2)	4.5×10^+3^(30)								
6075.9(20	272.0	0.15(3)	130(30)								
6126.3(15	218.1	15.1(2)	2.3(1)								
6243(20)	100.3	1.34(10)	83(7)								

In Table 9, E_α_’s, I_α_’s and their associated uncertainties for ^217^At were measured in [41], except for E_α_’s = 5500 and 5530, which were measured from the α-γ coincidence spectrum in [42]. For ^217^Fr and ^217^Ac, they were measured in [42] and [29], respectively.

The isomeric state energy levels (E_level_), their percentage decay by isomeric transition (% IT), their J^π^, and their measured half-lives for ^217^Bi, ^217^Ac and ^217^Pa, respectively, are listed in Table 10.

**Table 10:** Isomeric states and their properties for ^217^Bi, ^217^At and ^217^Ac.

^217^Bi [27]				^217^Ac [15]				^217^Pa [17]			
E_level_	%IT	J^π^	*T*_*1/2*_	E_level_	%IT	J^π^	*T*_*1/2*_	E_level_	%IT	J^π^	*T*_*1/2*_
1436+x	100	(25/2-)	3.0(2) μs	1146.7(8)	≥ 99.7	17/2-		1850	27(4)		1.2(2) ms
				1149.2(10)	≥ 98.3	15/2-					
				1498.2(9)	≥ 99.0	19/2-	8(2) ns				
				1528.5(9)	≥ 99.6	21/2-	<10ns				
				2013	≥ 95.7(10)	(29/2+)	740(40) ns				

In Table 10, the half-live T_1/2_ for the E = 1436+x in ^217^Bi was measured in [27], whereas for ^217^Ac, it was measured from the γγ prompt coincidences in [15]. Meanwhile, for ^217^Pa, it was calculated as an unweighted average of 1.6(10) ms [32], 0.(6) ms [43], 1.5(2) ms [44] and 1.5(+9–4) ms [34], respectively. B(E2) was calculated for ^217^Bi from the systematics of neighboring nuclides and ranges from 0.00062(3) for x = 20 keV for the isomeric states 1436+x keV to 0.00044(2) for x = 90 keV [3]. In addition, an octupole deformation has been noticed in ^217^Fr from the large value of B(E1)/B(E2) [17].

**Fig 1:**
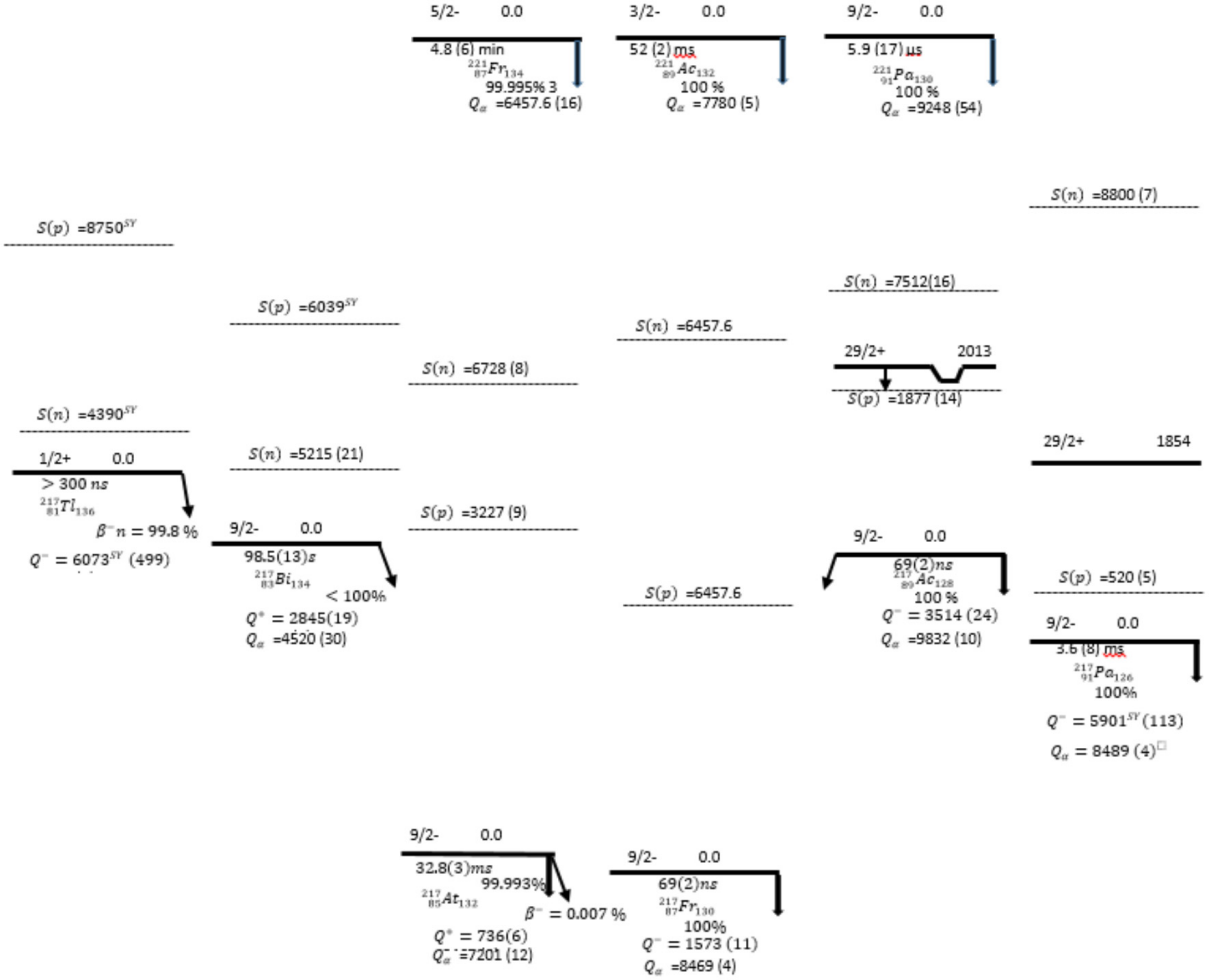
A skeleton scheme for A = 217: Odd- proton nuclei.

**Fig 2:**
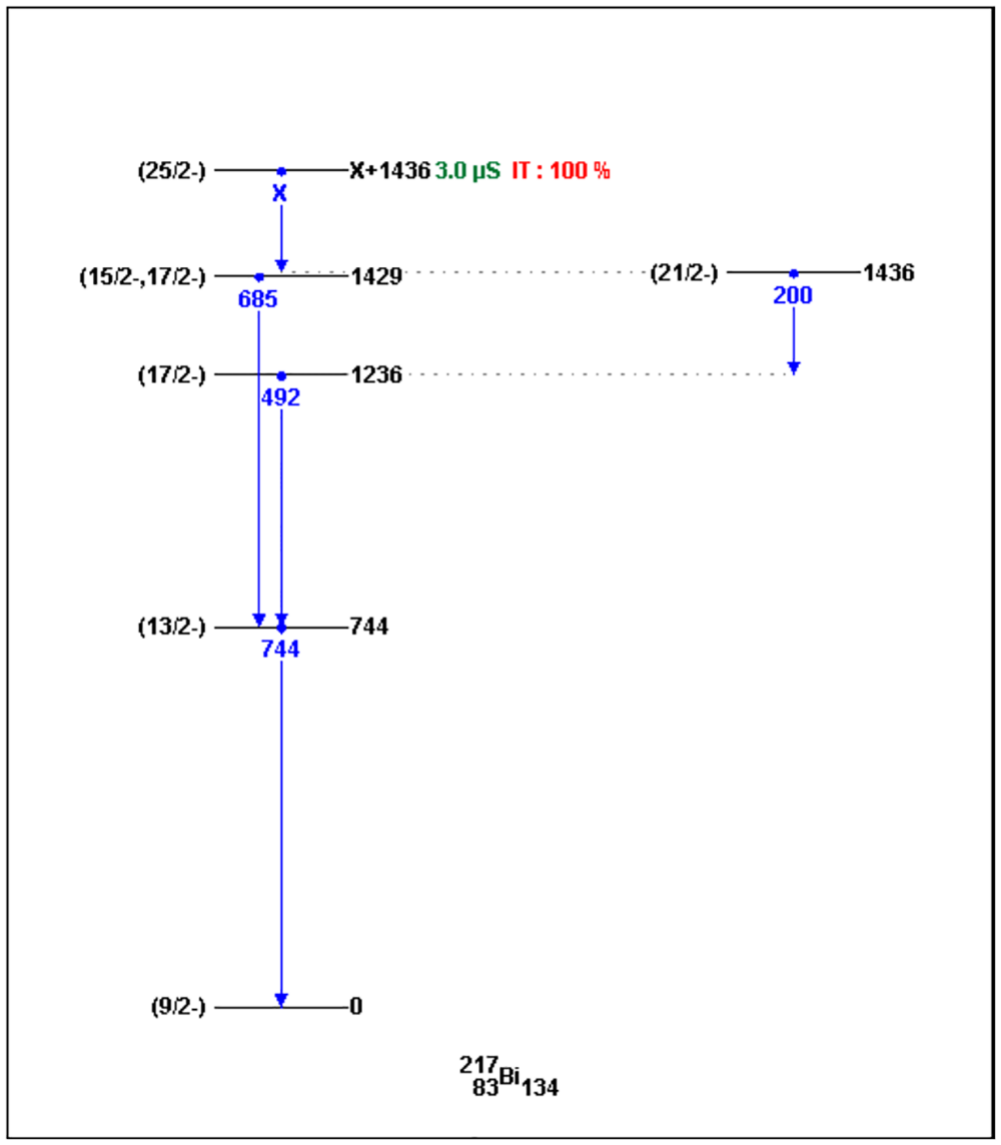
The complete decay scheme of ^217^Bi based on the current evaluation. Gamma transition energy is in blue color, the black lines are for the level energies of ^217^Bi, whereas, the green color is for the half- lives and red color is for the decay type.

**Fig 3:**
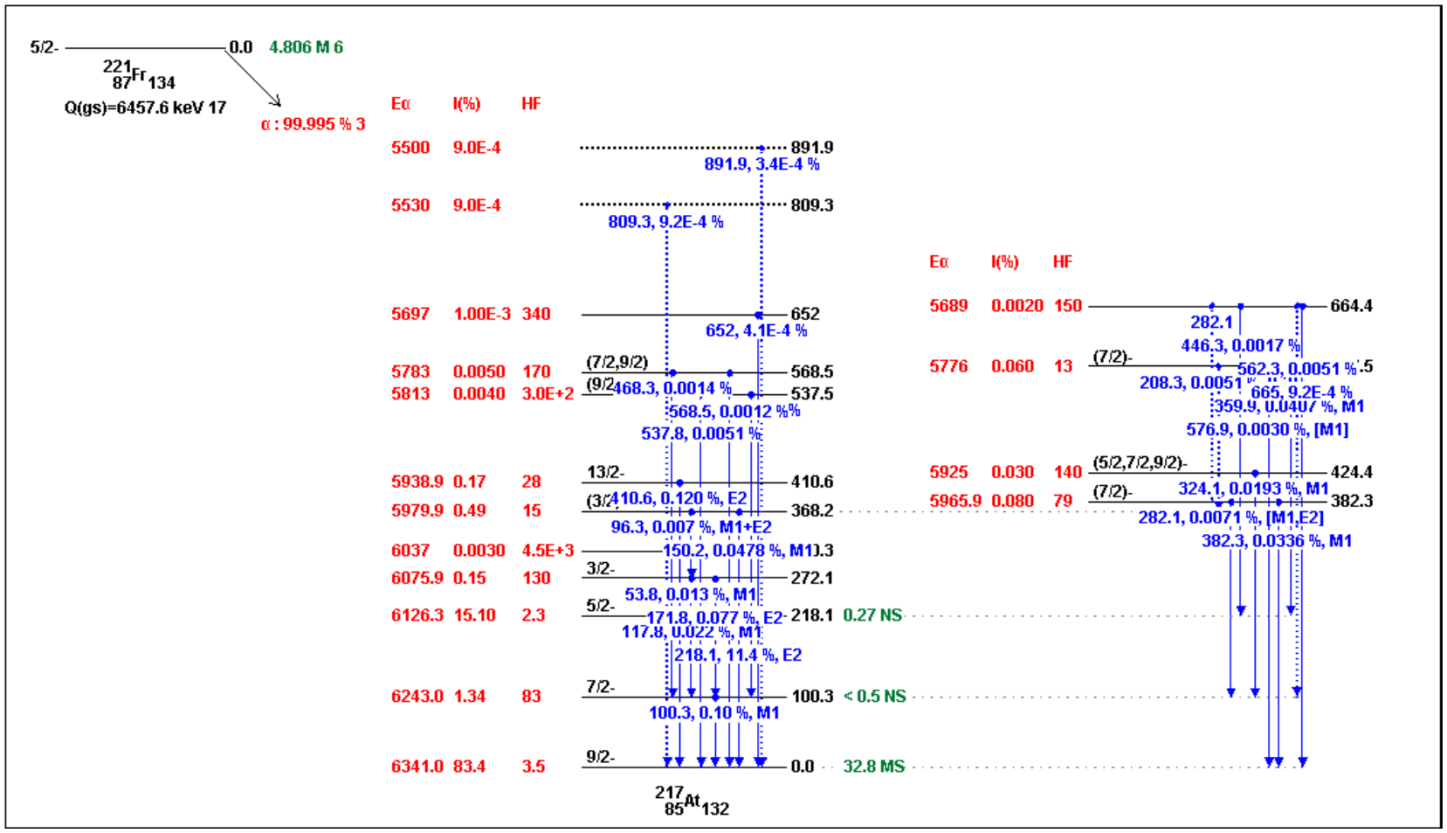
The complete decay scheme of ^217^At based on the current evaluation. Gamma transition energy and multipolarities are in blue color, the black lines are for the level energies of ^217^At, whereas, the green color is for the half- lives and red color is for the α- decay properties (E_α_, I_α_ and *HF*).

**Fig 4:**
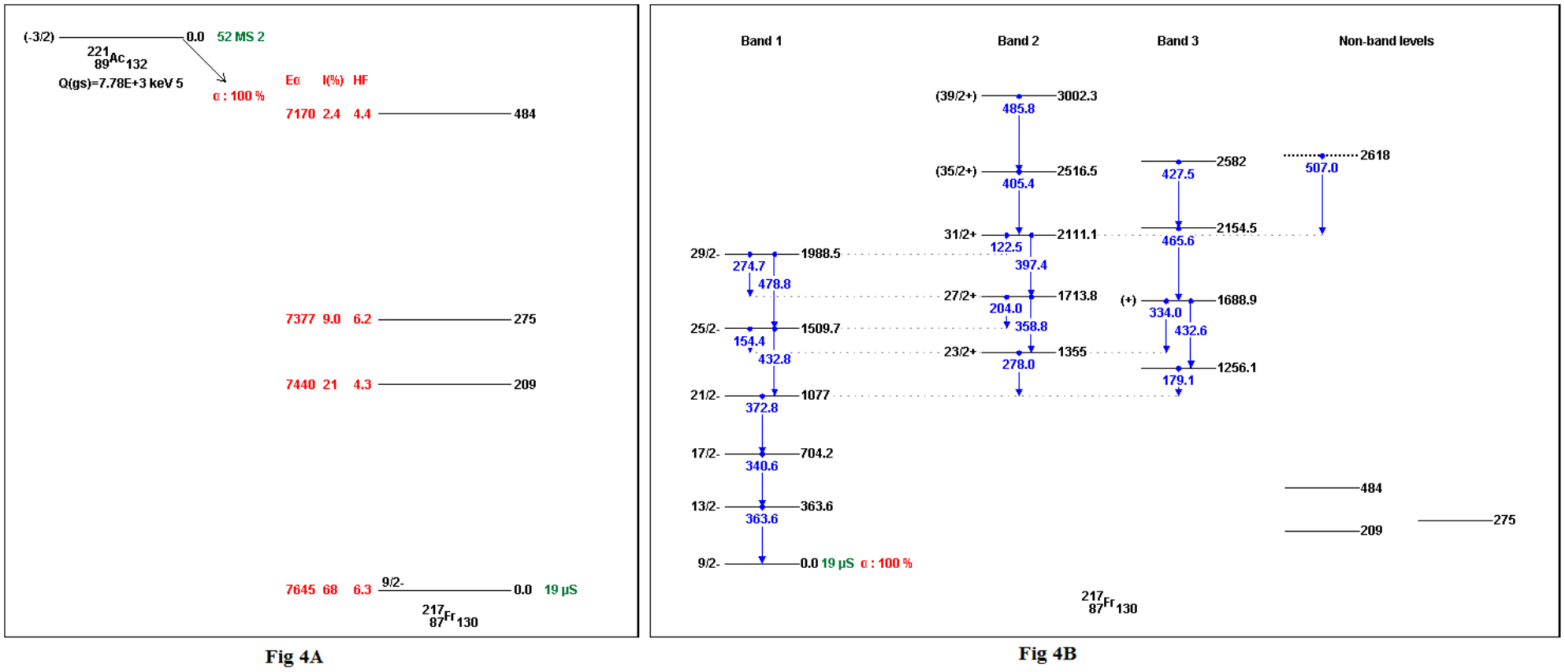
The complete decay scheme of ^217^Fr based on the current evaluation. A) the α- decay properties (E_α_, I_α_ and *HF*) in red color. B) Gamma transition energy is in blue color, the black lines are for the level energies of ^217^ Fr, whereas, the green color is for the half- lives.

**Fig 5:**
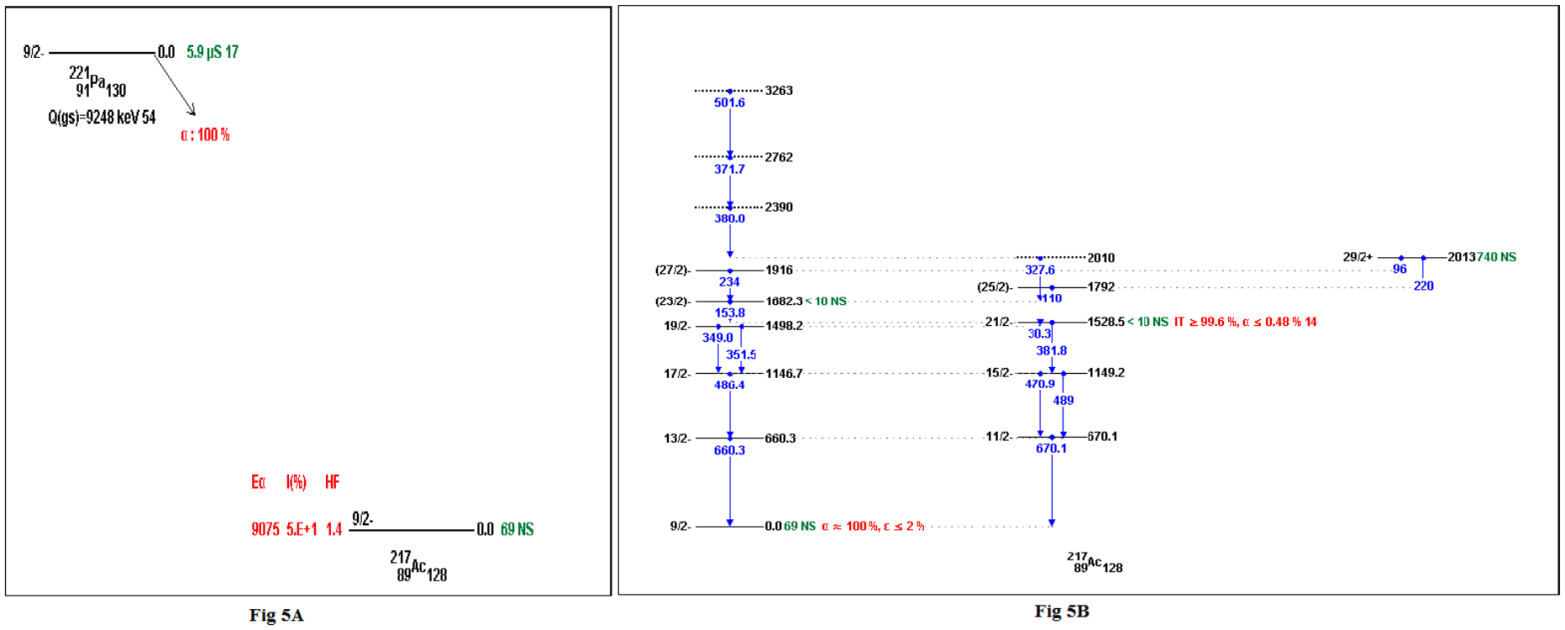
The complete decay scheme of ^217^Ac based on the current evaluation. A) the α- decay properties (E_α_, I_α_ and *HF*) in red color. B) Gamma transition energy is in blue color, the black lines are for the level energies of ^217^ Ac, whereas, the green color is for the half- lives.

**Fig 6:**
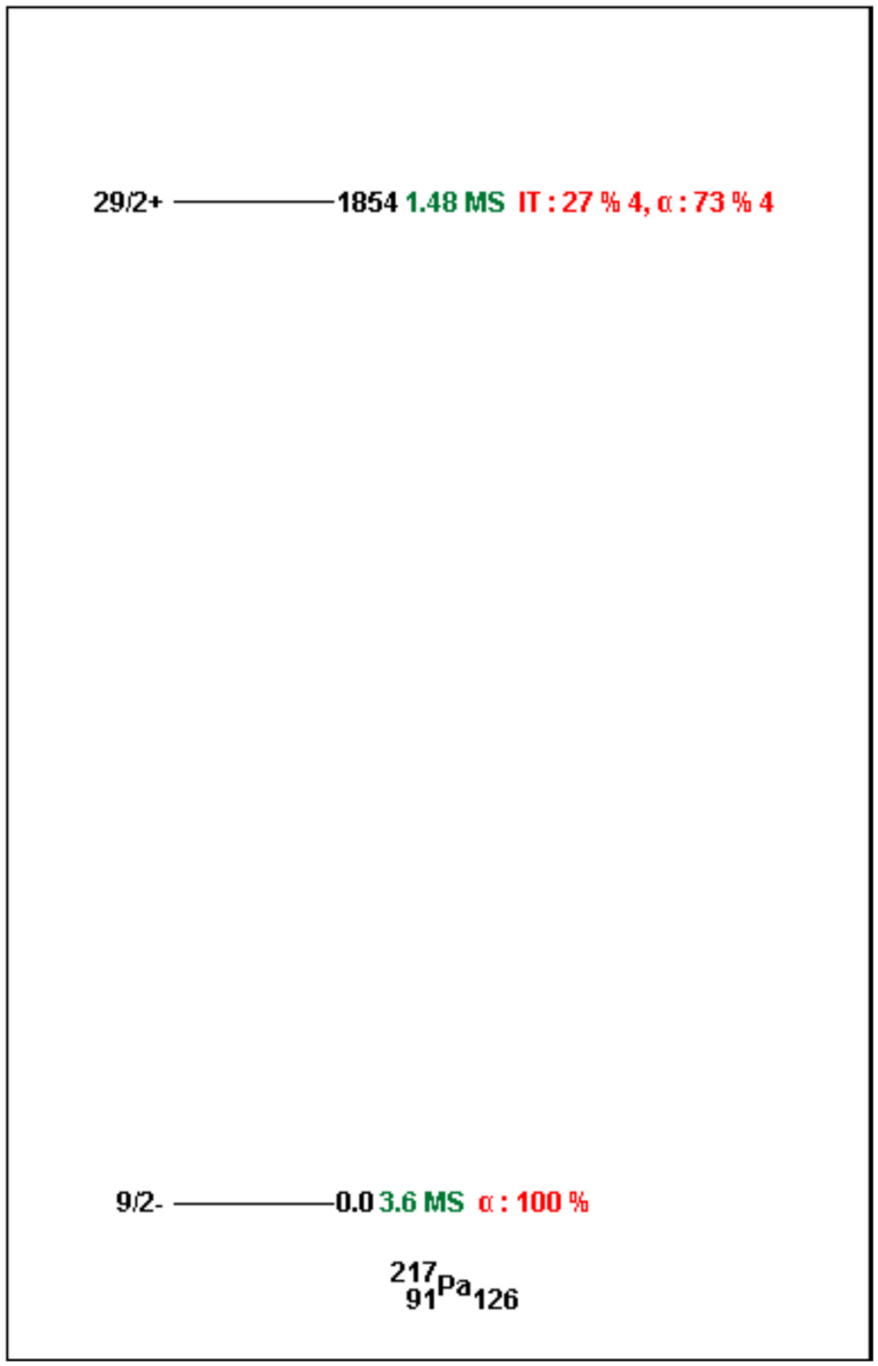
The complete decay scheme of ^217^Pa based on the current evaluation.

Skeleton schemes for ^217^Tl, ^217^Bi, ^217^At, ^217^Fr, ^217^Ac and ^217^Pa are shown in Fig 1. The complete decay schemes of ^217^Bi, ^217^At, ^217^Fr, ^217^Ac and ^217^Pa based on the current evaluation (S1–S12 Datasets) are shown in Figs 2–6, respectively. Gamma transition energies with their emission probabilities, spins and parities for energy levels, hindrance factors for α- decays and band structures are included in the figures. Whereas, Intensities I(γ+ce) are expressed per 100 parent decays.

## Conclusions

The evaluated nuclear structure data files (ENSDF) for nuclides of odd-proton numbers among the mass chain A = 217 (Tl81217,Bi83217, At85217, Fr87217, Ac89217 and Pa91217) have been updated in the present work. All literature works have been studied until the cut-off date April 2015. The half-lives, the Q (α) and Q (β) values, the total conversion electrons as well as the K-Shell to L-Shell, L-Shell to M-Shell and L-Shell to N-Shell conversion electron ratios have been reevaluated and adopted in the present work. Moreover, an updated skeleton decay scheme for each of the above nuclei has been presented here. In addition, the updated decay schemes include the assigned multipolarities, the emission probabilities, gamma-transitions and the evaluated decay hindrance factor (HF) for α-decays whenever possible. The new ENSDF datasets for the above nuclides have been sent to the National Nuclear Data Center (NNDC) at Brookhaven National Laboratory (BNL) for consideration of online publication.

## Supporting Information

S1 Dataset Adopted levels for ^217^Tl. (TXT)

S2 Dataset Adopted levels, Gammas for ^217^Bi. (TXT)

S3 Dataset ^98^E (^238^U, x)^217^Bi. (TXT)

S4 Dataset Adopted levels, Gammas for ^217^At. (TXT)

S5 Dataset ^221^Fr alpha decay .
(TXT)

S6 Dataset Adopted levels, Gammas for ^217^Fr. (TXT)

S7 Dataset ^221^Ac alpha decay. (TXT)

S8 Dataset ^210^Pb (^11^B, 4nγ) ^217^Fr. (TXT)

S9 Dataset Adopted levels, Gammas for ^217^Ac. (TXT)

S10 Dataset (HI, xnγ) ^217^Ac. (TXT)

S11 Dataset ^221^Pa alpha decay. (TXT)

S12 Dataset Adopted levels, Gammas for ^217^Pa. (TXT)
